# Visual Cortex Plasticity: A Complex Interplay of Genetic and Environmental Influences

**DOI:** 10.1155/2012/631965

**Published:** 2012-07-18

**Authors:** José Fernando Maya-Vetencourt, Nicola Origlia

**Affiliations:** ^1^Laboratorio di Neurobiologia, Scuola Normale Superiore, Piazza dei Cavalieri 7, 56126 Pisa, Italy; ^2^Institute of Neuroscience, CNR, Via Moruzzi 1, 56124 Pisa, Italy

## Abstract

The central nervous system architecture is highly dynamic and continuously modified by sensory experience through processes of neuronal plasticity. Plasticity is achieved by a complex interplay of environmental influences and physiological mechanisms that ultimately activate intracellular signal transduction pathways regulating gene expression. In addition to the remarkable variety of transcription factors and their combinatorial interaction at specific gene promoters, epigenetic mechanisms that regulate transcription have emerged as conserved processes by which the nervous system accomplishes the induction of plasticity. Experience-dependent changes of DNA methylation patterns and histone posttranslational modifications are, in fact, recruited as targets of plasticity-associated signal transduction mechanisms. Here, we shall concentrate on structural and functional consequences of early sensory deprivation in the visual system and discuss how intracellular signal transduction pathways associated with experience regulate changes of chromatin structure and gene expression patterns that underlie these plastic phenomena. Recent experimental evidence for mechanisms of cross-modal plasticity following congenital or acquired sensory deprivation both in human and animal models will be considered as well. We shall also review different experimental strategies that can be used to achieve the recovery of sensory functions after long-term deprivation in humans.

## 1. Introduction

As development proceeds, the nervous system begins to process information from the external world thus creating neuronal representations of the environment, which are continuously modified by sensory experience. Interactions with the external world, mediated by sensory input, update and modify the structural and functional architecture of the central nervous system, particularly during short-term periods in early life (known as critical periods) as experience drives the consolidation of synaptic circuitries [1, 2]. However, the reorganization of neuronal representations continues in adult life, as, for instance, in response to learning, loss of sensory input, trauma, or disease. The basis of the continuous and dynamic change in neuronal representations of sensory functions is a hot area of current neuroscience research with potential applications in the fields of neuronal regeneration, brain plasticity, and repair.

Long regarded as a rather static and unchanging structure, the adult brain has increasingly been recognized as a system that retains a degree of plasticity that allows for a rewiring of neural networks over the entire life course. The visual system is a classical neurobiological paradigm in this context. Structural and functional modifications of neural circuitries in the visual cortex relay on a complex interplay between long-distance neuromodulatory systems [3–17], together with experience-dependent neuronal activity mediated by local inhibitory and excitatory neurotransmission [18–24], neurotrophic factors [15, 16, 25–29], extracellular matrix molecules [30–35], hormones [36, 37], and endocannabinoids [38]. A complex interaction between these physiological processes seems to set in motion intracellular signal transduction pathways [39–41] that eventually promote the expression of transcription factors and downstream target genes that mediate phenomena of plasticity. The result is a highly dynamic architecture of the brain that is continuously modified by sensory experience. In fact, the reorganization of cortical circuitries persists late in life, at least to some extent (for review see [42]). Achieving a fundamental understanding of physiological processes that lie behind neuronal plasticity may be of clinical relevance in pathological states where the reorganization of neuronal networks would be beneficial in adult life. 

The importance of sensory experience in development of the human brain is well exemplified by cases of strabismic or anisometric children that underwent no clinical treatment during early development. In either pathological condition proper visual experience is altered, causing a marked impairment of normal visual functions (amblyopia) that is irreversible if not treated before 8 years of age [43, 44]. A similar phenomenon is described by clinical observations of children born with congenital cataracts, a pathological condition in which the lens of the eye becomes milky and therefore no longer permits images to form on the retina [45, 46]. Although amblyopia can be prevented by different therapeutic strategies that restore the formation of proper retinal images or by eye patching in early life, such treatments are normally ineffective in adults [47, 48]. Therefore, the recovery of normal visual functions after long-term sensory deprivation has long been a subject of study with the prospect of finding therapies for human amblyopia in adulthood. These observations indicate that proper sensory experience during early stages of development is necessary for normal sensory perception and also point towards the enhancement of neuronal plasticity as a strategy for brain repair in adult life. 

## 2. Early Sensory Deprivation and Visual Cortical Plasticity

Although intrinsic factors drive the initial assembly of synaptic circuitries in the nervous system, neuronal networks are shaped by experience during early postnatal life. Once basic patterns of neural connections are formed, an experience-dependent organization of eye-specific inputs is the major mechanism by which synaptic connectivity is achieved in the developing visual cortex.

 The monocular deprivation paradigm is a classic model to assess neuronal plasticity in the visual system. Pioneering electrophysiological studies in cats and monkeys clearly demonstrated that short periods of visual deprivation by unilateral eye closure during early development cause structural and functional modifications in visual cortical circuitries. Visual cortex responsiveness actually shifts in favour of the normal eye after monocular deprivation during the critical period [49–52]. Furthermore, the deprived eye becomes amblyopic: its visual acuity (spatial resolution) and contrast sensitivity are markedly impaired [53–55]. At structural level, unilateral eyelid suture causes a reduction in the arborisation of geniculocortical terminals that serve the deprived eye, which parallels an increased spread of terminals serving the open eye [51] and is consistent with the fact that monocular deprivation impairs the spatial resolution of geniculate neurons [56]. Because this type of deprivation does not cause amblyopia in adulthood, this early temporal window characterized by an enhanced brain susceptibility to sensory experience is a typical example of a critical period. It is interesting to note that most cortical cells remain responsive to both eyes following a period of binocular deprivation in juvenile age. Thus, it appears that afferents from the two eyes compete for cortical territory and that the relative amount of activity in the two eyes determines the outcome of this competitive process.

## 3. Nonvisual Components of the Environment Alter Visual System Development

Another classical paradigm used to assess the impact of experience in the functional maturation of the visual system is dark rearing (i.e., rearing animals in total darkness from birth). Total absence of visual experience delays the functional maturation of the striate cortex [57–59]. This event seems to be mediated by a downregulation of BDNF expression in early life [60], which results in a retarded maturation of GABAergic circuitries that control functional development of the visual system (for review see [20]). The spatial resolution of visual cortical neurons is actually reduced in dark-reared animals, this phenomenon being accompanied by longer latencies of responses to visual stimuli [58, 59]. Additionally, rearing animals in complete darkness extends the critical period far beyond its normal limits [61]. 

Although early studies of the visual system showed that sensory experience after eye opening is necessary for the functional maturation of the visual cortex [49–52], the notion that nonvisual components of the environment influence development of the visual system has been increasingly appreciated in the last few years. Experiments that combine dark rearing and electrophysiology as a functional readout, in transgenic mice overexpressing BDNF in forebrain regions, revealed a remarkable and unexpected finding: visual cortical neurons in these animals responded normally to visual stimuli, indicating that they could see well despite the lack of visual experience during the critical period [62]. These observations are in consonance with the fact that environmental enrichment, an experimental condition characterized by an increased exploratory behavior and sensory-motor stimulation, prevents the effects of dark rearing in the visual system [63]. This effect has been ascribed to an increased BDNF signaling and enhanced GABAergic inhibition during early stages of development [63]. Of note, environmental enrichment in normally reared animals accelerates the functional development of the visual cortex, this phenomenon being accompanied by alterations in the expression of BDNF and GABA synthesizing enzymes well before eye opening [64]. These findings further suggest that environmental influences on visual system development are, at least, partially independent of visual experience. 

## 4. Physiological Mechanisms That Regulate Developmental Plasticity in the Visual System

### 4.1. The Maturation of Inhibitory Circuitries Controls the Time Course of the Critical Period

The experience-dependent maturation of GABA-mediated inhibition during development establishes the beginning of the critical period for plasticity in the visual system [18–20]. This was demonstrated by seminal electrophysiological studies in transgenic mice that lack one isoform of the GABA synthesizing enzyme GAD65 and therefore show reduced levels of intracortical inhibition. No variation of visual cortex responsiveness was observed after monocular deprivation during early life in GAD65 transgenic animals, whereas enhancing inhibition by means of GABA-A receptor agonists rescued the impairment of plasticity [18, 19]. Therefore, a reduction of inhibitory transmission in early life halts the onset of the critical period for visual cortex plasticity (for review see [20]). A second inhibitory threshold that causes the end of the critical period is reached over postnatal development as well. Transgenic animals that overexpress BDNF in forebrain regions display an accelerated maturation of intracortical inhibitory circuitries, which, in turn, causes a precocious development of the visual system and therefore a fast end of the critical period for ocular dominance plasticity [29]. In summary, an initial threshold of inhibition triggers a sensitive period in which neuronal networks in the visual system are highly susceptible to sensory experience, whereas a second inhibitory threshold signals the end of this phase of enhanced plasticity. 

Inhibition triggers plasticity through GABA-A receptors containing the alpha-1 subunit [[21], reviewed in [20]]. These receptors are enriched at somatic synapses on pyramidal neurons made by large basket cells (a class of parvalbumin-positive GABAergic interneurons that extend horizontally across ocular dominance columns). Recent studies indicate that visual experience controls the time course of the critical period by promoting the transfer of the homeoprotein Otx2 from the retina to the visual cortex, where it appears to promote the maturation of parvalbumin-positive GABAergic interneurons [22]. Indeed, a reduction of inhibitory transmission is observed in visual cortical slices from Otx2-knockout animals, suggesting that Otx2 is a retinal messenger that triggers the critical period by enhancing levels of inhibition. Moreover, intracortical delivery of the recombinant Otx2 protein in animals before the onset of the critical period (in which no shift of ocular dominance is observed after eyelid suture due to low levels of inhibition) renders the visual cortex sensitive to monocular deprivation [22]. Accordingly, the impairment of plasticity in Otx2-knockout animals is rescued by enhancing GABA-A receptor currents by benzodiazepine treatments [22]. These findings suggest that visual experience signals the time course of the critical period by activating the retinogeniculocortical transfer of the protein Otx2 in the visual pathway. 

### 4.2. Extracellular Matrix Molecules Restrict Plasticity in the Developing Visual System

An emerging view in neuronal plasticity research is that the effects caused by early sensory experience in the remodeling of visual cortical circuitries are actively preserved throughout life by the late appearance of molecular factors in the extracellular milieu that restrict plasticity. The establishment of neuronal connectivity may be, at least in part, under control of structural factors such as myelin-associated proteins (NgR, PirB) and chondroitin sulphate proteoglycans (CSPGs), which all are inhibitory for axonal sprouting [30–32, 65, 66]. Different experimental findings support this notion. The maturation of intracortical myelination, for instance, correlates with the end of the critical period and ocular dominance plasticity persists well into adulthood in NgR-knockout mice [30]. Knockout animals lacking the NgR ligand Nogo-A also display plastic phenomena in adult life, thus confirming that NgR-dependent mechanisms restrict plasticity in the visual system. Additionally, the paired immunoglobulin-like receptor B (PirB) shows high affinity for Nogo-A, the signaling of which is inhibitory for axonal regeneration [31]. In keeping with this, PirB restricts ocular dominance plasticity in the developing visual cortex [32]. 

Likewise, the condensation of CSPGs around the soma and dendrites of parvalbumin-positive GABAergic interneurons parallels the time course of the critical period, whereas CSPGs degradation by exogenous administration of the enzyme chondroitinase-ABC reactivates visual cortex plasticity in the adult [34, 35]. This is consistent with the notion that removing extracellular matrix components that are inhibitory for axonal growth [65, 66] provides a permissive environment for structural plasticity (e.g., by modifying dendritic spine dynamics) and associated functional modifications in the visual cortex. It is important to remark that degradation of CSPGs may alter the ratio of inhibitory/excitatory transmission in the visual cortex as these glycoproteins condense in perineuronal nets (PNNs) mainly around parvalbumin-positive GABAergic interneurons. So far, however, the impact of chondroitinase-ABC treatment on the intracortical inhibitory/excitatory balance in the visual system remains to be explored. 

### 4.3. Long-Distance Neuromodulatory Systems Regulate Visual Cortical Plasticity

The major modulatory systems in the brain (i.e., adrenaline, noradrenaline, dopamine, acetylcholine, and serotonin) regulate complex functions of the central nervous system such as different forms of brain plasticity, cognitive processes, and behavior. Experience-dependent modifications of cortical circuitries are not determined solely by local correlations of electrical activity but are also influenced by attentional mechanisms. Sensory signals, for instance, promote marked modifications of neural circuitries mainly when animals attend to the sensory input and use this information for the control of behavior (reviewed in [67, 68]). Accordingly, early studies performed in kittens demonstrated that changes of visual cortical circuitries in response to experience are lessened when noradrenergic [3, 4], cholinergic [4, 5], and serotonergic [6–8] projections to the cortex are inactivated. Moreover, there is evidence that these neuromodulatory systems mediate forms of visual cortex plasticity late in life both in cats [9–13] and rodents [14–17]. 

Advances in the understanding of mechanisms by which neuromodulatory systems regulate experience-dependent plasticity derive from in vitro studies of synaptic plasticity. There is evidence that noradrenaline, acetylcholine, and serotonin modulate two different forms of activity-dependent synaptic modifications: long-term potentiation (LTP) and long-term depression (LTD). In the visual system, LTP and LTD can be induced by different patterns of electrical stimulation. Brief and strong episodes of high frequency stimulation promote LTP while prolonged low-frequency stimulation yields LTD. In the rodent visual cortex, upon administration of noradrenaline and acetylcholine, weaker tetanic stimulation is required to induce LTP and shorter episodes of low frequency stimulation are needed to drive LTD [69, 70]. Likewise, serotonin facilitates the induction of both LTP and LTD in layer IV of the kitten visual system [8]. These findings are consistent with a role for neuromodulatory systems as enabling factors for visual cortical plasticity and indicate that activation of noradrenergic, cholinergic, and serotonergic receptors lowers the threshold of activity required for the induction of LTP and LTD. Intracellular mechanisms whereby neuromodulatory systems facilitate these forms of synaptic plasticity have been subject of extensive study. The induction of LTP and LTD requires the activation of N-methyl-D-aspartate (NMDA) receptors together with a postsynaptic rise in intracellular calcium. The available evidence is consistent with a model in which the magnitude and duration of the calcium signal determines the magnitude of the synaptic modification [71]. Brief and large calcium influxes induce LTP, whereas smaller and prolonged calcium increases yield LTD. Of note, receptors of these three major neuromodulatory systems are able to activate the IP3 second messenger pathway, which can induce calcium release from intracellular stores and therefore modulate plasticity. Because the intracortical inhibitory/excitatory balance regulates experience-dependent plasticity in the visual system (for review see [20, 24]), the neuromodulators-mediated fine-tuning of the inhibitory/excitatory ratio is likely to play a key role in the induction of plastic phenomena. Accordingly, it has been recently demonstrated that the enhanced signaling of either serotonin or acetylcholine shifts the inhibitory/excitatory balance in favour of excitation in the rodent visual cortex [72, 73]. 

The critical period for the induction of LTP evoked by stimulation of thalamocortical connections almost overlaps the duration of the critical period for ocular dominance plasticity. In addition to thalamocortical connections, LTP and LTD can be elicited by stimulation of intrinsic connections both during postnatal development and in adulthood and these forms of plasticity are only partially dependent on NMDA receptors [74]. LTP in layer II/III cells is facilitated by concomitant application of muscarinic and noradrenergic agonists but not by the single application of each neurotransmitter [69]. Accordingly, exogenous administration of acetylcholine in visual cortical slices induces LTP through stimulation of muscarinic acetylcholine receptors (mAChRs) [75]. Moreover, LTP is impaired in visual cortex slices from transgenic mice with reduced cortical cholinergic innervation due to the expression of an anti-NGF antibody [76]. Exogenous application of acetylcholine, however, rescues LTP suggesting an essential role of this neurotransmitter in cortical synaptic plasticity. In agreement with this notion, immune depletion of basal forebrain cholinergic neurons with IgG-192 saporin impairs LTP in the visual cortex [77]. Furthermore, it has been reported that visual cortex LTP and LTD are modulated by the activation of different mAChRs [78]. Using single and double muscarinic receptor knock-out mice, it has been demonstrated that normal LTP is expressed when M2 and M4 are coactivated while LTD relays more on M1 and M3 receptor. Moreover, while prolonged low-frequency stimulation normally induces LTD, it does yield LTP in M1-knockout animals. These findings suggest that the direction of synaptic plasticity can be modulated by the combined activity of different mAChRs, possibly by regulating the threshold for synaptic modification. 

## 5. The Reinstatement of Plasticity in the Adult Visual System 

**Figure 1:**
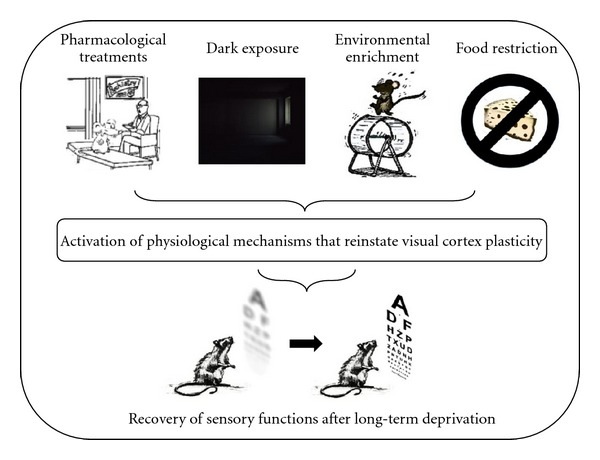
Experimental paradigms that restore neuronal plasticity in adult life. Environmental enrichment [17, 81, 82], long-term fluoxetine administration [15, 16], visual deprivation by dark exposure [79, 80], food restriction [36], and IGF-1 treatment [83] are noninvasive experimental approaches that promote adult visual cortical plasticity by altering the balance of inhibition and excitation in the visual system. The potential for the reactivation of plasticity caused by some of these paradigms to promote the recovery of sensory functions after long-term sensory deprivation has been reported using amblyopia as a paradigmatic model [15, 36, 80, 81, 83].

The identification of molecular and cellular mechanisms at the basis of brain plasticity and the enhancement of plasticity as a strategy for brain repair in adult life are hot areas of current neuroscience research. As previously described, the developmental maturation of intracortical inhibitory circuitries causes the end of plasticity in the visual system (reviewed in [20]). In keeping with this notion, it is possible to restore plasticity in adult life by reducing levels of inhibition. A direct demonstration that GABAergic signaling is a crucial brake limiting visual cortex plasticity derives from the observation that a pharmacological decrease of inhibitory transmission effectively restores ocular dominance plasticity in adulthood [23]. Accordingly, experimental paradigms such as dark exposure [79, 80], environmental enrichment [17, 81, 82], food restriction [36], long-term fluoxetine treatment [15, 16], and exogenous IGF-I administration [83] all promote plasticity late in life by reducing the intracortical inhibitory/excitatory ratio (Figure 1). This has prompted the search for endogenous factors with the potential to enhance plasticity in adult life by modulating the intracortical I/E balance.

**Figure 2:**
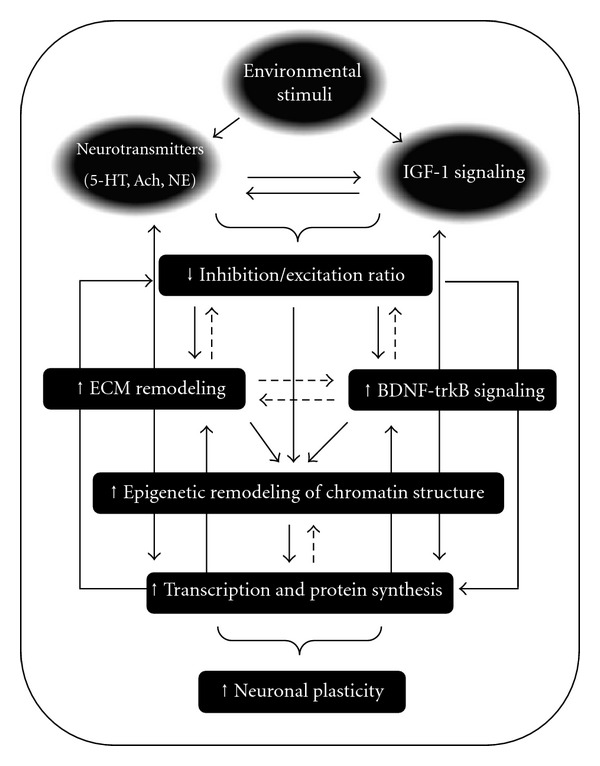
The reinstatement of ocular dominance plasticity in adulthood is associated with signal transduction pathways that involve the enhanced action of either neuromodulatory projection systems (e.g., serotonin and acetylcholine) or IGF-1 signaling, which all set in motion physiological processes that modulate the inhibitory/excitatory ratio in favour of excitation [14–17, 72, 73, 83]. A shift of the inhibitory/excitatory balance may directly activate intracellular mechanisms that eventually promote epigenetic modifications of chromatin structure (e.g., changes of DNA methylation patterns and/or posttranslational modifications of histones), which in turn allow for the expression of genes that act as downstream effectors of plastic phenomena in adult life. A pharmacological reduction of intracortical inhibition enhances plasticity while promoting the activity-dependent BDNF expression (unpublished data) and degradation of extracellular matrix (ECM) components that are inhibitory for plasticity [23]. BDNF-trkB signaling might upregulate the expression of additional genes associated with functional modifications in the visual cortex. Degradation of ECM components (e.g., CSPGs) may modify the inhibition/excitation ratio in the visual system. The interaction between BDNF-trkB signaling and ECM reorganization has yet to be explored. Continuous arrows represent established interactions between the molecular and cellular processes mentioned (boxes). Dashed lines represent interactions that remain to be ascertained.

Previous studies have demonstrated that the process of plasticity reactivation in the adult visual system is a multifactorial event that comprises the action of different cellular and molecular mechanisms, working in parallel or in series, the sum of which results in the activation of intracellular signal transduction pathways regulating the expression of plasticity genes [14–17, 34–36, 83–86], [for review see [42]]. In rodents, experimental paradigms based upon the enhancement of environmental stimulation levels, genetic manipulations, and pharmacological treatments have revealed that the enhanced action of either long-distance projection systems (e.g., serotonergic and cholinergic transmission) or IGF-I signaling seems to modulate the intracortical inhibitory/excitatory balance in favour of excitation [72, 73, 83], which in turn, sets in motion cellular and molecular events that eventually mediate the expression of genes associated with functional modifications in the adult visual system (Figure 2). The reinstatement of plasticity caused by enhanced serotonergic transmission, for instance, is mediated by 5-HT1_A_ receptors signaling and accompanied by increased BDNF expression [16]. This is paralleled by heightened histone acetylation status at the activity-dependently regulated BDNF promoter regions and by decreased expression of histone deacetylase enzymes (HDACs) [16]. In keeping with this, increasing histone acetylation levels by long-term treatment with HDACs inhibitors (e.g., trichostatin-A, valproic acid, and sodium butyrate) not only reinstates ocular dominance plasticity in adulthood [16, 84] but also promotes full recovery of visual functions in adult amblyopic animals [85]. Accordingly, environmental enrichment, long-term fluoxetine treatment, and food restriction all increase acetylation of histones in the hippocampus and cortex in adult life [16, 36, 87].

## 6. Structural Plasticity in the Visual Cortex

Experience-dependent functional modifications in the visual system are accompanied by a structural remodeling of synaptic connectivity, in terms of growth and loss of dendritic spines. Dendritic spines in pyramidal neurons are markedly sensitive to experience. Total lack of visual experience in early life induces modifications in spine morphology and density, both of which are partially reversible by light exposure [88]. Accordingly, monocular deprivation in early life alters the motility, turnover, number, and morphology of dendritic spines in the visual cortex [89–92]. 

Does structural plasticity contribute to experience-dependent modifications of neural circuitries? Structural plasticity in vivo studies, using two-photon imaging, indicate that dendritic spine dynamics is high during early postnatal life but decreases thereafter, in parallel to the time course of critical period plasticity over development (reviewed in [93]). This suggests that, despite the absence of large-scale structural remodeling, the reorganization of cortical connections in terms of growth and loss of dendritic spines may be the structural substrate for experience-dependent plasticity. Notably, chronic imaging experiments have recently demonstrated that changes in visual cortex responsiveness after monocular deprivation during the critical period correlate with dendritic spines structural modifications across the visual cortex, these two features being reversed when the deprived eye is reopened. After brief periods of monocular deprivation, spine turnover increases significantly, with a larger percentage of spines being lost rather than gained, whereas after a 24-hour period of recovery (visual experience) the total number of dendritic spines is reestablished [94]. Accordingly, increasing the density and dynamics of spines by intracortical infusion of the bacterial toxin CNF1 restores a degree of plasticity in the mature cortex that is similar to that observed during early postnatal life [95].

It is worth mentioning that new synapses formation may increase memory storage capacity of the brain and that new dendritic spines may serve as structural traces for earlier memories, enabling the brain for faster adaptations to similar future experiences [91, 96]. Recent experiments carried out using the monocular deprivation paradigm seem to confirm this notion. Modifications of dendritic spines caused by a first experience of unilateral eyelid suture persist even after restoration of binocular vision and may therefore be involved in the enhancement of plasticity observed after a second episode of visual deprivation [91]. 

The imaging studies mentioned above raise the question of whether structural modifications of dendritic spines represent functional changes of synaptic transmission. Electrophysiological experiments in hippocampal slice cultures indicate that AMPA- and NMDA-type glutamate receptor currents of newborn spines resemble those of mature synaptic contacts [97]. It has been recently demonstrated that dynamics of dendritic spine development regulates the stability of synaptic plasticity. The relationship between calcium influx and spine size actually determines the long-term synaptic stability and synaptic strength distribution in synapses of hippocampal CA3-CA1 pyramidal neurons [98]. 

## 7. Epigenetic Mechanisms of Plasticity

Long-term functional modifications of neural circuitries are mediated by a complex interplay between cellular and molecular mechanisms that activate intracellular signal transduction pathways regulating gene expression. Besides the remarkable diversity of transcription factors and their combinatorial interaction at gene promoter areas, the role of epigenetic mechanisms that control chromatin susceptibility to transcription in response to experience has been increasingly appreciated [99]. Growing experimental evidence indicates that chromatin structure is highly dynamic within the nervous system and that it is recruited as a target of plasticity-associated signal transduction pathways. The remodeling of chromatin structure is actively involved in activity-dependent neuronal plasticity in different brain areas via regulation of gene expression [100, 101]. 

Processes of chromatin remodeling that modulate gene transcription are conserved mechanisms by which the mammalian nervous system accomplishes adaptive behavioral responses upon environmental demands. In rodents, maternal care seems to influence behavioral and endocrine responses to stress in the offspring by modifying chromatin susceptibility to gene expression. It has been demonstrated that rat pups that are most licked and groomed during postnatal development, display, later in life, better performance in tests of learning and memory than pups that get licked less [102]. Interestingly, high licking/grooming pups are less anxious than low licked/groomed counterparts, and this behavior seems to be epigenetic rather than inherited. The genetic reprograming by maternal behavior actually emerges over the first week of postnatal life and can be reversed by cross-fostering: if high licking/grooming dams rear the biological offspring of low licking/grooming ones, the offspring actually behave as high licking/grooming pups [103]. At molecular level, maternal care promotes the expression of the glucocorticoid receptor, this phenomenon being accompanied by a decreased DNA methylation status at the glucocorticoid receptor gene promoter area in the hippocampus [103]. Moreover, changes in the pattern of DNA methylation correlate with modifications at the level of histones, as high licking/grooming pups show enhanced levels of acetylation of lysine 9 on histone 3 (H3K9), which is a marker of gene transcription activation. This is consistent with the observation that increasing histones acetylation by hippocampal infusion of the histone deacetylase (HDAC) inhibitor Trichostatin-A in low licking/grooming pups changes the methylation pattern to that of pups brought up by high licking/grooming dams. Furthermore, low licking/grooming pups treated with Trichostatin-A are also less anxious than vehicle-treated counterparts and show no difference at behavioral level as compared to high licking/grooming pups [103]. These findings illustrate the notion that sensory experience in early life drives epigenetic mechanisms of neuronal plasticity that underlie behavior. 

Similarly, phenomena of plasticity in the visual cortex of cats and rodents during the critical period require the activation of different intracellular protein kinases (e.g., PKA, ERK1/2, and CamKII) [39–41]. The activation of these intracellular signal transduction pathways promotes the upregulation of transcription factors that, in turn, mediate gene expression. A very well-known activity-dependent mechanism is the activation of the transcription factor CREB, which triggers the expression of genes under control of the cAMP-response element (CRE) promoter, thus allowing phenomena of plasticity to occur [104, 105]. These plastic events involve processes of chromatin remodeling. Visual experience during early life promotes modifications of chromatin structure that are permissive for transcription, whereas a developmental downregulation of histone post-translational modifications regulates the closure of the critical period in the mouse visual system [84]. Accordingly, directly increasing acetylation of histones by long-term treatment with HDACs inhibitors effectively reactivates plasticity in the adult visual system [16, 84, 85].

## 8. Short Noncoding mRNAs and the Regulation of Plasticity

In addition to the function of transcription factors and modifications of chromatin structure, growing experimental evidence supports a critical role for short noncoding RNAs (microRNAs), which interact with and control translation of mRNA targets, in the regulation of gene expression patterns at the basis of plastic phenomena in the mammalian nervous system [106, 107]. MicroRNAs are powerful regulators of gene expression and act by binding to the 3′-untranslated region (3′-UTR) of the target mRNA, making it possible for a single microRNA to control expression of multiple genes that posses the same sequence in this region of the mRNA. 

The brain-specific microRNA, miR-134, for instance, has been found to localize in the synaptodendritic compartment of rat hippocampal neurons and negatively regulates the size and density of dendritic spines [108]. This effect seems to be achieved by miR-134 posttranscriptional inhibition of the mRNA that encodes the protein kinase, LimK1, which controls dendritic spine development. This was demonstrated by refined experiments in which miRNA-134 was overexpressed in hippocampal neurons together with constructs expressing either a wild-type Limk1 mRNA or a mutant Limk1 mRNA that is incapable of interacting with miRNA-134. The study of spine morphology revealed that coexpression of the wild-type Limk1 mRNA, which is still subject to miRNA-134 translational inhibition, caused a decreased spine size phenotype. In contrast, expression of the mutant Limk1 mRNA that is incapable of interacting with miRNA-134 rescued the spine defect [108]. Hence, both overexpression of miRNA-134 and disruption of Limk1 function lead to decreased spine size. Accordingly, exposure of neurons to neurotrophins such as BDNF, which promotes synaptic development, maturation, and plasticity, relieves miRNA-134 inhibition of Limk1 mRNA translation [108]. These findings indicate that miR-134 disrupts dendritic and synaptic development by repressing Limk1 mRNA translation. 

Recent experimental evidence points toward a key role for another microRNA, miRNA-132, as a molecular transducer of neuronal plasticity. It has been reported that synaptic activity promotes a CREB-dependent miRNA-132 expression and that miRNA-132 induction is necessary for the activity-dependent dendritic growth [109]. The effect of miRNA-132 on dendrite morphology seems to be mediated by the activation of the Rac1-PAK actin-remodeling pathway that is due to the miRNA-132 translational inhibition of the mRNA that encodes the protein p250GAP, which is a Rho family GTPase activating protein [110]. This is consistent with the observation that overexpression of miRNA-132 in neuronal cultures promotes neuronal morphogenesis [111] and is in line with the fact that transgenic mice overexpressing miRNA-132 in forebrain regions display an increased spine density [112]. Interestingly, downstream target genes regulated by miRNA-132 mediate phenomena of chromatin remodeling and protein translation in the suprachiasmatic nucleus of rodents [113], these two molecular processes being critically involved in the occurrence of neuronal plasticity. 

Electrophysiological studies have addressed the role of microRNAs as mediators of synaptic plasticity at hippocampal level. LTP of synaptic transmission in the dentate gyrus of rodents is accompanied by an upregulation of miRNA-132 [114], while its overexpression in cortical neurons regulates short-term plasticity [115]. More recently, evidence for the role of miRNA-132 as a mediator of visual cortical plasticity has been obtained in vivo by using the experience-dependent monocular deprivation paradigm. It has been reported that miRNA-132 is rapidly upregulated after eye opening in normally reared animals. This phenomenon is delayed by dark rearing, whereas monocular deprivation in early life results in a decrease of miRNA-132 expression. Remarkably, reducing miRNA-132 neonatal expression by lentiviral infection [116] or counteracting the miRNA-132 downregulation in response to monocular deprivation [117] effectively prevents ocular dominance plasticity in the developing visual system. These data highlight the notion that optimal physiological levels of miRNA-132 are critical for plasticity to occur during the critical period. Interestingly, neonatal blockade of miRNA-132 expression in early life results in an immature state of dendritic spines [116], whereas counteracting the miRNA-132 downregulation after monocular occlusion increases the percentage of mushroom-stubby dendritic spines that represent the more stable state of spines [117]. These findings suggest that miRNA-132 is a molecular transducer of the action of visual experience on developing visual circuitries, possibly acting through modulation of dendritic spines plasticity [118]. 

## 9. Cross-Modal Plasticity: Adaptive Reorganization of Neural Networks in Early Life

Sensory deprivation in one modality during early stages of development can have marked effects on the development of the remaining modalities. This phenomenon is known as cross-modal plasticity and is particularly epitomized by cases of congenital blindness or deafness from birth. In such instances, processes of cross-modal plasticity strengthen other sensory systems to compensate for the lack of vision or hearing.

Although clinical studies of deaf and blind humans have clearly demonstrated increased functional capabilities and compensatory expansion in the remaining sensory modalities (reviewed in [119]), the neurological bases for these plastic phenomena remain poorly understood. It has been reported that congenitally blind subjects show better sound localization abilities as compared to sighted individuals [120] and display better two-point tactile discrimination skills as well [119]. Studies that combine Braille reading and functional brain imaging revealed that early blind individuals show a strong activation of the occipital cortex during the reading task [121, 122], this phenomenon being independent of attentional mechanisms [122]. Activation of the visual cortex has also been reported during the tactile object recognition task [123]. Remarkably, the inactivation of the visual cortex by means of transcranial electrical stimulation in blind people during Braille reading not only distorts tactile perceptions of blind subjects but also induces errors in Braille reading [124]. Furthermore, the visual cortex in blind subjects is recruited by language processing (e.g., semantic and phonological tasks) [125–127]. There is also evidence that congenital blindness enables visual circuitries to contribute to olfactory processing [128].

The question of whether there is a critical period for cross-modal plasticity has also been addressed by examining the activation of visual cortical areas by Braille reading in early and late-onset blind individuals. It has been reported that visual cortex responsiveness to somatosensory stimuli (Braille reading) is higher in congenitally blind and early-onset subjects as compared to the late-onset blind group [129–132]. These data indicate that there is a critical period for the visual cortex to be recruited to a role in the processing of somatosensory information, which does not extend beyond 14 years of age in humans. An important question that remains to be answered concerns structural and functional mechanisms whereby phenomena of cross-modal plasticity occur. It has been reported that stabilization of long-range cortico-cortical connections between sensory modalities may mediate, at least, some aspects of these plastic phenomena [133]. Such cross-modal connections have been described in several species. Anatomical evidence for direct connections between primary auditory cortex and primary visual cortex in adult monkeys has been previously reported [133].

Phenomena of cross-modal plasticity have also been observed in the brain of deaf subjects. Functional magnetic resonance imaging studies have demonstrated that early deaf individuals use the primary auditory cortex alongside the visual system when they observe sign language [134]. Although there is no hearing component to sign language, the auditory cortex is instead used to assist with visual and language processing. The effects of cochlear implants also provide another strategy to assess cross-modal plasticity in the deaf. Early deaf individuals, but not late-onset deaf subjects, actually display impairments in their ability to process language using a cochlear implant in adult life as the auditory cortex has been reshaped to deal with visual information and therefore it cannot deal as well with the new sensory input that the implant provides [135]. 

A recent series of experiments using environmental enrichment [136] as a strategy to investigate the influence of sensory experience on brain development, and in particular the somatosensory stimulation in terms of body massage, provide evidence that mechanisms of cross-modal plasticity are likely to underlie the beneficial effects of enhancing somatosensory activity in development of another sensory modality, the visual system [137]. It has been reported that an enriched environment accelerates the structural and functional development of the rodent visual system [64] and that enriching the environment in terms of tactile stimulation (body massage with a soft toothbrush) in rat pups effectively mimics the effects of enrichment on visual system development [137]. The massage protocol in the offspring of rats accelerated the maturation of visual functions and increased circulating levels of IGF-1, whereas antagonizing IGF-1 signaling by systemic injections of JB1 (IGF-1 receptor antagonist) prevented the effects of massage [137]. Remarkably, enriching the environment in terms of body massage in human preterm infants accelerates the maturation of the visual system as indicated by an enhanced development of spatial acuity, this effect being correlated to high IGF-1 serum levels [137]. Taken together, these findings indicate that processes of cross-modal plasticity may be involved in the effects caused by environmental enrichment in visual cortex development and portray the well-characterized visual system as a model to understand the functional integration of two or more sensory modalities.

## 10. Can We Treat Human Amblyopia in Adult Life?

**Figure 3:**
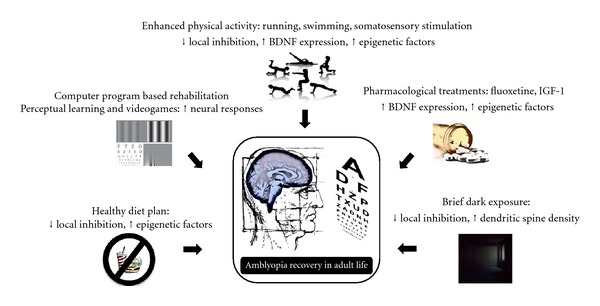
Potential strategies for the treatment of human amblyopia in adult life. The recent findings that environmental enrichment [17, 81, 82, 137, 138], long-term administration of fluoxetine [15, 16], dark exposure [79, 80], food restriction [36], and IGF-1 signaling [83] all promote full recovery of visual acuity and binocularity in adult amblyopic animals, emphasize the potential of different pharmacological and/or behavioral interventions as complementary strategies for current therapies of human amblyopia in adult life. In particular, an enhanced sensory-motor activity together with a healthy diet planning, brief periods of visual deprivation by dark exposure, and pharmacological treatments (long-term antidepressant treatment or exogenous IGF-1 administration) may enhance plasticity by shifting the I/E ratio while increasing BDNF expression and epigenetic factors. These therapeutic interventions could be coupled to video game playing or computer-program-based training of the amblyopic eye in order to rescue normal visual functions after long-term sensory deprivation in humans.

The potential clinical application of experimental strategies that promote plasticity in the adult visual system has long been explored with the prospect of treating human amblyopia, a pathological condition that arises from an abnormal visual experience during development and is refractory treatment in the adult (for review see [43, 47]). The data on animal models reported along this paper suggest that an enhanced sensory-motor activity [17, 137, 138], a healthy diet planning [36], brief periods of dark exposure [79, 80], fluoxetine administration [15, 16], and IGF-1 treatment [83] may be used as complementary strategies to current therapies for human amblyopia (Figure 3). Clinical trials that include pharmacological and behavioral interventions by long-term fluoxetine treatment together with a computer-program-based training of the amblyopic eye to rescue amblyopia in adult life are underway in Finland and New Zealand (L. Maffei, personal communication. http://www.hermopharma.com/news-a-publication/120-first-patients-completed-the-amblyopia-phase-2a-study).

In this context, perceptual learning has long been used to improve spatial acuity in adult amblyopic patients (for review see [139]). Systematic training of patients with unilateral amblyopia (secondary to strabismus and anisometropia) in simple visual tasks revealed a 2-fold increase of contrast sensitivity and improved performance in letter-recognition tests [140]. Likewise, Snellen acuities in anisometric amblyopes improved after intensive training in a Vernier acuity task. Moreover, video game playing seems to promote a significant rescue of visual functions in adult amblyopic patients. Playing video games (both action and nonaction games) for a short period of time using the amblyopic eye results in a substantial improvement in a wide range of fundamental visual functions, including visual acuity, positional acuity, spatial attention, and stereopsis [141].

The improvement of performance seen in perceptual learning is proportional to the number of trials taken, although performance eventually reaches an asymptote of no further progress [142]. Unfortunately, the extent to which acuity improvements occur is limited by the task specificity of perceptual learning [143]. It is worth mentioning that in most instances of perceptual learning, attention to the trained stimulus is necessary for improvements of vision to occur [144]. This observation is particularly important as it epitomizes the role of long-distance neuromodulatory systems in the physiological state of arousal, which regulates mechanisms of attention and information processing that may contribute to functional changes of neural circuitries in the adult brain. This points toward the possibility to pharmacologically enhance plasticity as a strategy for brain repair in a variety of pathological states where reorganization of neuronal networks would be beneficial in adult life. This, for instance, could facilitate restructuring of mature circuitries impaired by damage or disease. Long-term pharmacologically induced serotonergic transmission actually enhances the effects of rehabilitation in the recovery from motor deficits after ischemic stroke in humans [145].
